# Prevalence and Associated Factors of Anxiety and Depression Among Primary Caregivers of Children With Haematological Malignancies: A Cross-sectional Study

**DOI:** 10.62641/aep.v54i2.2195

**Published:** 2026-04-15

**Authors:** Jiao Li, Jianghong Wu, Yuhan Huang, Yun Zhuang

**Affiliations:** ^1^Department of Hematology, Children's Hospital of Soochow University, 215003 Suzhou, Jiangsu, China; ^2^Pediatric Clinical Medical College, Children's Hospital of Soochow University, 215003 Suzhou, Jiangsu, China; ^3^Nursing Department, Children's Hospital of Soochow University, 215003 Suzhou, Jiangsu, China

**Keywords:** haematological malignancies, caregivers, anxiety, depression, social support, symptom burden

## Abstract

**Background:**

Primary caregivers of children with hematological malignancies endure immense physical and psychological stress, however their mental health status remains under-recognized in clinical settings. This study aimed to investigate the prevalence of anxiety and depression among these caregivers and to identify their independent associated factors, providing evidence for targeted nursing interventions.

**Methods:**

A cross-sectional survey was conducted involving 200 primary caregivers recruited from the Department of Haematology, Children's Hospital of Soochow University. Demographic and clinical data were collected alongside psychological assessments using the Hospital Anxiety and Depression Scale (HADS), the Memorial Symptom Assessment Scale (MSAS), and the Multidimensional Scale of Perceived Social Support (MSPSS). Univariate analyses and multivariable logistic regression models were employed to determine the associations between potential predictors and psychological distress outcomes.

**Results:**

The study revealed a substantial burden of psychological morbidity. Specifically, 52.00% of caregivers exhibited anxiety and 42.50% showed symptoms of depression. Multivariable analysis further identified distinct risk profiles for each condition. Anxiety was independently associated with shorter time since diagnosis (odds ratio (OR) = 0.66, 95% confidence interval (CI): 0.47–0.92, per 6 months), greater child symptom burden (OR = 1.22, 95%: CI 1.03–1.46, per 10 points increase), and social support (OR = 0.48, 95% CI: 0.32–0.71, per 10 points). Conversely, depression was significantly associated with sociodemographic factors including educational level (OR = 0.34, 95% CI: 0.13–0.89, for college degree or above) and single marital status (OR = 3.97, 95% CI: 1.35–11.69), in addition to symptom burden (OR = 1.55, 95% CI: 1.25–1.91, per 10 points) and social support (OR = 0.31, 95% CI: 0.19–0.50, per 10 points). Furthermore, sensitivity analyses highlighted that the frequency recent hospitalizations were consistently associated with higher levels of both anxiety and depression.

**Conclusions:**

Caregivers of children with haematological malignancies experience exhibits a high prevalence of anxiety and depression. Anxiety appears to be more closely related to acute clinical stressors and temporal factors, whereas depression is more closely related to persistent social and demographic disadvantages. Effective management of paediatric symptoms and the strengthening of multi-dimensional social support systems are essential. Future interventions should be tailored to the specific risk profiles of caregivers to improve their overall well-being.

## Introduction

Paediatric haematological malignancies, particularly acute lymphoblastic leukaemia, are among the most common childhood cancers and usually require prolonged multi-phase treatment [1, 2]. Management typically involves prolonged chemotherapy, repeated hospitalizations, and intensive medical follow-up [2]. Although advances in treatment have substantially improved survival rates, both the disease and its treatment continue to impose considerable and enduring burdens on affected families [3, 4]. As the central figures in the child’s care, primary caregivers play a pivotal role throughout treatment and recovery, and their physical and psychological well-being is closely linked to the quality of care provided to the child [5, 6].

During the treatment of paediatric haematological malignancies, caregivers frequently often provide continuous bedside care and participate actively in treatment-related decision-making and emotional support [5, 7]. These sustained responsibilities place caregivers under significant psychological strain. Previous studies have demonstrated that anxiety and depression are common among parents of children with cancer, although the reported prevalence varies across studies. A meta-analysis reported estimated pooled prevalence rates of 21% for anxiety and 28% for depression, and both exceeding those observed in population controls [8]. A nationwide cohort study further indicated that parents of children with cancer had a higher risk of mental health disorders than parents of cancer-free children [9]. Data specifically focusing on haematological malignancies highlight a more prolonged trajectory of distress due to repeated relapses and intensive chemotherapy regimens. Persistent psychological distress in parents may impair family functioning and is associated with poorer quality of life in children during cancer treatment [10, 11].

Furthermore, caregiver anxiety and depression are influenced by multiple interrelated factors [12]. Existing evidence suggests that lower educational attainment, greater financial strain, and limited social support are common demographic and socioeconomic factors associated with psychological distress among parents of children with cancer [13, 14]. From a clinical perspective, distress tends to be greater around the time of diagnosis, and a higher symptom burden in the child has been associated with poorer parental psychosocial outcomes [15, 16]. These factors may operate simultaneously, and their associations with caregiver psychological distress should be interpreted within a multidimensional framework.

While the psychological burden on cancer caregivers is documented globally, research specifically addressing the unique trajectory of paediatric haematological malignancies in China remains fragmented [17]. Most existing studies focus on a limited range of predictors, often failing to capture the complex interplay between clinical stressors and social resources [18]. This study addresses this gap by integrating child symptom burden, caregiver socioeconomic status, and multidimensional social support within a single analytical model. Such a comprehensive framework is necessary to determine the independent association of each factor and to understand the specific correlates of anxiety and depression among caregivers.

Therefore, we conducted a cross-sectional study to evaluate the prevalence of anxiety and depression among primary caregivers and to identify their distinct risk profiles. By analysing clinical, socioeconomic, and psychosocial variables simultaneously, we aimed to uncover the specific correlates of psychological distress in the high-intensity caregiving environment of paediatric haematology. Clarifying these relationships holds substantial clinical significance. The findings will provide a robust evidence base for early psychological screening and help healthcare professionals develop tailored nursing interventions. This approach ensures that limited medical resources are effectively directed toward the most vulnerable caregiver subgroups, ultimately improving the overall quality of family-centred care.

## Methods

### Study Design and Setting

This cross-sectional study using a convenience sampling method and was conducted from February to September 2025 in the inpatient haematology ward of Children’s Hospital of Soochow University. The study was approved by the Ethics Committee of Children’s Hospital of Soochow University (2025CS190). Written informed consent was obtained from all participants. All data were used exclusively for research purposes and were handled confidentially with appropriate anonymization. This study was conducted in accordance with the principles of the Declaration of Helsinki.

### Participants

Primary caregivers of hospitalized children in the haematology department were recruited. A primary caregiver was defined as the family member who provided the greatest amount of day-to-day care during hospitalization and was most involved in treatment-related communication and caregiving decisions. To ensure data independence, only one primary caregiver was recruited for each child. Inclusion criteria were as follows: (1) the child was diagnosed with a hematologic malignancy; (2) the child was aged 14 years or younger; (3) the caregiver was a parent or an immediate family member (e.g., grandparent) providing unpaid care; (4) caregiving duration exceeded 3 months; (5) written informed consent was obtained. Exclusion criteria were: (1) a history of psychiatric disorders, current psychological treatment, or recent major stressful events that could substantially affect emotional status; (2) the presence of severe congenital diseases unrelated to the haematological system in the child.

The sample size was calculated using the estimation formula for a single population proportion. Assuming an expected prevalence of 50 percent to maximize variance alongside a margin of error of 0.08 and a 95 percent confidence level, the minimum required sample size was calculated to be 150. Factoring in a potential nonresponse and invalid rate of 20 percent, the target sample size was set to at a minimum of 188 participants. A total of 205 questionnaires were distributed during the study period. Ultimately 200 valid questionnaires were included in the final analysis. This resulted in a questionnaire missing and invalid rate of 2.44 percent. Five questionnaires were excluded. Three due to excessive missing key items beyond the predefined acceptable threshold. One because the caregiving duration was less than 3 months. The final one because the caregiver was currently receiving psychological treatment.

### Outcomes

Caregiver anxiety and depression were assessed using the Anxiety and Depression subscales of the Hospital Anxiety and Depression Scale (HADS), including the Anxiety (HADS-A) and Depression (HADS-D) subscales [19]. The Chinese version of this scale has been extensively validated and demonstrates robust construct validity and reliability across diverse clinical populations. Each subscale contains 7 items scored from 0 to 3, yielding a total score ranging from 0 to 21. A subscale score greater than 7 was used to indicate the presence of anxiety or depressive symptoms. Symptom severity was categorized into three levels including 0 to 7 for normal, 8 to 10 for borderline, and 11 to 21 for abnormal. In the present study, the scale demonstrated high internal consistency. The Cronbach α coefficient was 0.94 for the anxiety subscale and 0.92 for the depression subscale (**Supplementary Table 1**).

### Explanatory Variables

Explanatory variables included caregiver characteristics, child clinical characteristics, and socioeconomic factors. Caregiver characteristics comprised age, sex, relationship to the child, educational level, marital status, and employment status. Child clinical characteristics included age, sex, diagnosis, time since diagnosis, treatment stage, and number of hospitalizations in the past 3 months. Child symptom burden was assessed using the Chinese version of the Memorial Symptom Assessment Scale for children aged 7 to 12 (MSAS 7–12) [20]. This instrument evaluates multiple dimensions of symptoms, including symptom occurrence and, where applicable symptoms, severity and distress. Although the MSAS 7–12 was originally designed for school-aged children, it has been widely validated for proxy reporting by caregivers of paediatric cancer patients. Given that some children too young for self-administration, we utilized a uniform proxy-reporting approach where primary caregivers evaluated the child’s symptoms over the past week. Higher scores on the recorded symptom dimensions indicate greater symptom burden. Socioeconomic factors included monthly household income, insurance type, distance to the hospital, and perceived social support. Perceived social support was assessed using the total score of the Multidimensional Scale of Perceived Social Support (MSPSS), ranging from 12 to 84, with higher scores indicating greater perceived support [21].

### Data Collection Procedures

During hospitalization, trained investigators distributed questionnaires on-site and collected them immediately upon completion. Caregivers self-administered the demographic and socioeconomic items, HADS-A, HADS-D, and MSPSS. Child symptom burden was assessed using the MSAS 7–12. Primary caregivers completed the scale as proxy respondents based on the child’s clinical symptoms and distress during the previous week. Clinical data were extracted from electronic medical records, including diagnosis, time since diagnosis, treatment stage, and number of hospitalizations in the past 3 months. Data were checked for logic and internal consistency after entry. No missing data were identified.

### Statistical Analysis

Statistical analyses were performed using SPSS version 27.0 (IBM Corp., Armonk, NY, USA). Normality of continuous variables was assessed using the Shapiro-Wilk test. Since the data followed a non-normal distribution, variables were reported as the median and the first and third quartiles (Q1, Q3). Categorical variables are expressed as frequencies and percentages. Demographic and clinical characteristics of caregivers and children were described first, followed by the prevalence and severity distribution of caregiver anxiety and depression. Univariable comparisons were conducted by stratifying caregivers according to anxiety status and depression status, respectively. Continuous variables were compared using Mann-Whitney U test, and categorical variables were compared using Chi-square test. Multivariable logistic regression models were fitted separately for anxiety and depression as dependent variables. To enhance clinical interpretability, continuous independent variables were rescaled: time since diagnosis was analysed in 6-month increments, and symptom burden and social support scores were analysed in 10-point increments. Covariates entered the models if they showed *p* values less than 0.20 in univariable analyses or were considered clinically important based on prior knowledge, using the Enter method. Specifically, child age and diagnosis were retained in the multivariable models as essential control variables to ensure the independent effects of psychosocial factors were accurately estimated. Multicollinearity was assessed using variance inflation factors (VIF) and tolerance. Model calibration was evaluated using the Hosmer-Lemeshow goodness-of-fit test, and discrimination was assessed using the area under the receiver operating characteristic (ROC) curve (AUC). To assess the robustness of our results, we performed a sensitivity analysis by replacing caregiver-reported symptom scores with the frequency of hospitalizations in the past 3 months. This variable was selected as an objective proxy for disease severity and treatment intensity. Unlike subjective symptom ratings, hospitalization frequency provides a tangible measure of clinical instability. Comparing these two distinct dimensions of illness burden helps confirm that the identified associations with caregiver distress were consistent regardless of the measurement approach. All tests were two-sided, and statistical significance was set at *p* < 0.05.

## Results

### Demographic and Clinical Characteristics

A total of 200 primary caregivers were included in this study. The median age of the caregivers was 37.00 years. The majority were female, accounting for 67.50% of the sample. Mothers constituted the largest proportion of caregivers at 66.50%. Most participants were married. Regarding education, 39.00% had a high school education or equivalent, followed closely by those with a college degree or higher. Employment data showed that 41.50% held full-time jobs, while 34.50% were unemployed or had resigned to provide care. In terms of socioeconomic factors, the monthly family income predominantly ranged from 3000 to 8000 Chinese Yuan (CNY), equivalent to approximately United States dollar (USD) 435 - 1,160 based on an exchange rate of 1 USD = 6.8974 CNY, and more than half of the children were covered by resident health insurance. The median age of the children was 6.00 years, and 54.50% were male. Acute lymphoblastic leukaemia was the most frequent diagnosis, accounting for 69.50% of the cases. The median time since diagnosis was 8.00 months, and nearly half of the children in the maintenance treatment stage. In addition, the median number of hospitalizations for children in the last three months was 1.00. Detailed demographic and clinical characteristics are presented in Table 1.

**Table 1.** **Demographic and clinical characteristics of caregivers and children**.

Variables		Median (Q1, Q3)/n (%)
Caregiver characteristics		
Age (years)		37.00 (33.00, 42.00)
Sex		
	Male	65 (32.50)
	Female	135 (67.50)
Relationship with the child		
	Father	49 (24.50)
	Mother	133 (66.50)
	Grandparents	18 (9.00)
Educational level		
	Junior high school and below	48 (24.00)
	High school or equivalent	78 (39.00)
	College/bachelor’s degree or above	74 (37.00)
Marital status		
	Married	174 (87.00)
	Divorced/Widowed/Unmarried	26 (13.00)
Employment status		
	Full-time job	83 (41.50)
	Part-time/Temporary work	48 (24.00)
	Unemployed/Resigned care	69 (34.50)
Child clinical characteristics		
Age (years)		6.00 (3.00, 9.25)
Sex		
	Male	109 (54.50)
	Female	91 (45.50)
Diagnosis		
	ALL	139 (69.50)
	AML	32 (16.00)
	Lymphoma	29 (14.50)
Time since diagnosis (months)		8.00 (4.00, 14.00)
Treatment stage		
	Maintenance	98 (49.00)
	Induction/Consolidation	86 (43.00)
	Relapse/Palliative	16 (8.00)
Hospitalizations in last 3 months		1.00 (0.00, 2.00)
Symptom scores		48.30 (33.00, 65.80)
Socioeconomic factors		
Family income (CNY / USD per month)		
	<3000 / <435	50 (25.00)
	3000–8000 / 435–1160	103 (51.50)
	>8000/ >1160	47 (23.50)
Insurance type		
	Self-pay	17 (8.50)
	Resident insurance	117 (58.50)
	Employee/Commercial	66 (33.00)
Distance to hospital		
	Local city	59 (29.50)
	Intra-provincial	84 (42.00)
	Inter-provincial	57 (28.50)
Social support scores		55.00 (48.00, 62.00)

Continuous variables are presented as median (Q1, Q3). Categorical variables are reported as frequency (percentage). ALL, acute lymphoblastic leukaemia; AML, acute myeloid leukaemia; CNY, Chinese Yuan; USD, United States dollar; Q1, 1st quartile; Q3, 3rd quartile. Currency conversion was based on the March 2026 representative exchange rate of 1 USD = 6.8974 CNY. Converted U.S. dollar values are provided for reference only.

### Prevalence and Severity of Anxiety and Depression

The median anxiety score among primary caregivers was 8.00, and the median depression score was 7.00. These median scores are close to the clinical diagnostic threshold, indicating a poor overall mental status among the caregivers. Furthermore, the results indicated that 52.00% of the caregivers experienced anxiety symptoms. Regarding severity, 27.00% of the caregivers were classified as having abnormal anxiety levels, while 25.00% were in the borderline range. The overall prevalence of depression was 42.50%. Specifically, 20.50% of the caregivers showed abnormal levels of depression, and 22.00% were classified as borderline. Detailed scores, prevalence, and severity distributions of anxiety and depression are presented in Table 2.

**Table 2.** **Prevalence and severity of anxiety and depression among caregivers**.

Variables		Median (Q1, Q3)/n (%)
Anxiety scores		8.00 (4.75, 11.00)
Depression scores		7.00 (3.00, 9.25)
Anxiety		
	Yes	104 (52.00)
	No	96 (48.00)
Depression		
	Yes	85 (42.50)
	No	115 (57.50)
Severity of anxiety		
	Normal	96 (48.00)
	Borderline	50 (25.00)
	Abnormal	54 (27.00)
Severity of depression		
	Normal	115 (57.5)
	Borderline	44 (22.00)
	Abnormal	41 (20.50)

Continuous variables are presented as median (Q1, Q3). Categorical variables are reported as frequency (percentage). Q1, 1st quartile; Q3, 3rd quartile.

### Univariate Analysis of Anxiety Status

Univariate analysis demonstrated significant differences between the anxiety and non-anxiety groups regarding caregiver age (*p* = 0.045), time since diagnosis (*p* < 0.001), treatment stage (*p* = 0.003), and the frequency of hospitalizations in the preceding three months (*p* < 0.001). Specifically, compared to the non-anxiety group, caregivers in the anxiety group were older, and their children had a shorter duration since diagnosis, were more likely to be in the induction or consolidation phase, and experienced more frequent hospitalizations. Furthermore, significant differences were observed in symptom scores (*p* < 0.001) and social support scores (*p* < 0.001); the anxiety group reported a higher symptom burden and significantly lower levels of social support. No statistically significant differences were identified in other characteristics such as sex, education level, family income, or insurance type. Detailed comparisons are presented in Table 3.

**Table 3.** **Univariate comparisons of participant characteristics by anxiety status**.

Variables			Anxiety (n = 104)	Non-anxiety (n = 96)	Statistic	*p*
Caregiver characteristics						
	Age (years)		38.00 (33.75, 43.00)	36.00 (33.00, 40.00)	Z = –2.00	0.045
	Sex				χ^2^ = 0.44	0.506
		Male	36 (34.62)	29 (30.21)		
		Female	68 (65.38)	67 (69.79)		
	Relationship with the child				χ^2^ = 0.76	0.683
		Father	26 (25.00)	23 (23.96)		
		Mother	67 (64.42)	66 (68.75)		
		Grandparents	11 (10.58)	7 (7.29)		
	Educational level				χ^2^ = 4.40	0.111
		Junior high school and below	25 (24.04)	23 (23.96)		
		High school or equivalent	47 (45.19)	31 (32.29)		
		College/bachelor’s degree or above	32 (30.77)	42 (43.75)		
	Marital status				χ^2^ = 0.41	0.522
		Married	92 (88.46)	82 (85.42)		
		Divorced/Widowed/Unmarried	12 (11.54)	14 (14.58)		
	Employment status				χ^2^ = 0.14	0.933
		Full-time job	42 (40.38)	41 (42.71)		
		Part-time/Temporary work	25 (24.04)	23 (23.96)		
		Unemployed/Resigned care	37 (35.58)	32 (33.33)		
Child clinical characteristics						
	Age (years)		6.00 (3.00, 10.00)	6.00 (3.75, 9.00)	Z = –0.01	0.990
	Sex				χ^2^ = 0.43	0.510
		Male	59 (56.73)	50 (52.08)		
		Female	45 (43.27)	46 (47.92)		
	Diagnosis				χ^2^ = 0.17	0.918
		ALL	72 (69.23)	67 (69.79)		
		AML	16 (15.38)	16 (16.67)		
		Lymphoma	16 (15.38)	13 (13.54)		
	Time since diagnosis (months)		6.00 (4.00, 11.00)	10.00 (6.00, 15.25)	Z = –3.61	<0.001
	Treatment stage				χ^2^ = 11.66	0.003
		Maintenance	39 (37.50)	59 (61.46)		
		Induction/Consolidation	54 (51.92)	32 (33.33)		
		Relapse/Palliative	11 (10.58)	5 (5.21)		
	Hospitalizations in last 3 months		2.00 (1.00, 2.00)	1.00 (0.00, 2.00)	Z = –3.47	<0.001
	Symptom scores		55.05 (37.08, 71.90)	40.90 (30.43, 58.02)	Z = –3.31	<0.001
Socioeconomic factors						
	Family income (CNY / USD per month)				χ^2^ = 0.54	0.762
		<3000 / <435	27 (25.96)	23 (23.96)		
		3000–8000 / 435–1160	51 (49.04)	52 (54.17)		
		>8000/ >1160	26 (25.00)	21 (21.88)		
	Insurance type				χ^2^ = 1.02	0.601
		Self-pay	9 (8.65)	8 (8.33)		
		Resident insurance	64 (61.54)	53 (55.21)		
		Employee/Commercial	31 (29.81)	35 (36.46)		
	Distance to hospital				χ^2^ = 0.75	0.686
		Local city	28 (26.92)	31 (32.29)		
		Intra-provincial	46 (44.23)	38 (39.58)		
		Inter-provincial	30 (28.85)	27 (28.12)		
	Social support scores		52.00 (47.00, 58.25)	58.00 (51.75, 64.00)	Z = –3.76	<0.001

Continuous variables are presented as median (Q1, Q3). Categorical variables are reported as frequency (percentage). ALL, acute lymphoblastic leukemia; AML, acute myeloid leukemia; CNY, Chinese Yuan; USD, United States dollar; Q1, 1st quartile; Q3, 3rd quartile. Currency conversion was based on the March 2026 representative exchange rate of 1 USD = 6.8974 CNY. Converted U.S. dollar values are provided for reference only.

### Univariate Analysis of Depression Status

Univariate analysis demonstrated statistically significant differences between the depression and non-depression groups regarding educational level (*p* = 0.001), marital status (*p* = 0.035), time since diagnosis (*p* = 0.002), treatment stage (*p* = 0.010), and hospitalizations in the preceding three months (*p* < 0.001). Specifically, caregivers in the depression group were less likely to hold a college degree or above and more likely to be divorced, widowed, or unmarried compared to the non-depression group. Clinically, children in the depression group had a shorter time since diagnosis, were more frequently in the induction or consolidation phase, and experienced higher hospitalization rates. Moreover, significant differences were observed in child symptom scores (*p* < 0.001) and social support scores (*p* < 0.001), with the depression group reporting a higher symptom burden and significantly lower levels of social support. No significant differences were identified for other variables such as caregiver age, employment status, or household income. Detailed comparisons are presented in Table 4.

**Table 4.** **Univariate comparisons of participant characteristics by depression status**.

Variables			Depression (n = 85)	Non-depression (n = 115)	Statistic	*p*
Caregiver characteristics						
	Age (years)		37.00 (34.00, 43.00)	37.00 (32.00, 41.00)	Z = –1.00	0.316
	Sex				χ^2^ = 2.69	0.101
		Male	33 (38.82)	32 (27.83)		
		Female	52 (61.18)	83 (72.17)		
	Relationship with the child				χ^2^ = 1.19	0.551
		Father	23 (27.06)	26 (22.61)		
		Mother	53 (62.35)	80 (69.57)		
		Grandparents	9 (10.59)	9 (7.83)		
	Educational level				χ^2^ = 13.79	0.001
		Junior high school and below	24 (28.24)	24 (20.87)		
		High school or equivalent	42 (49.41)	36 (31.30)		
		College/bachelor’s degree or above	19 (22.35)	55 (47.83)		
	Marital status				χ^2^ = 4.43	0.035
		Married	69 (81.18)	105 (91.30)		
		Divorced/Widowed/Unmarried	16 (18.82)	10 (8.70)		
	Employment status				χ^2^ = 1.83	0.400
		Full-time job	37 (43.53)	46 (40.00)		
		Part-time/Temporary work	23 (27.06)	25 (21.74)		
		Unemployed/Resigned care	25 (29.41)	44 (38.26)		
Child clinical characteristics						
	Age (years)		6.00 (3.00, 10.00)	6.00 (3.00, 8.50)	Z = –0.87	0.386
	Sex				χ^2^ = 0.23	0.630
		Male	48 (56.47)	61 (53.04)		
		Female	37 (43.53)	54 (46.96)		
	Diagnosis				χ^2^ = 2.03	0.362
		ALL	55 (64.71)	84 (73.04)		
		AML	17 (20.00)	15 (13.04)		
		Lymphoma	13 (15.29)	16 (13.91)		
	Time since diagnosis (months)		6.00 (4.00, 11.00)	10.00 (5.00, 15.00)	Z = –3.05	0.002
	Treatment stage				χ^2^ = 9.25	0.010
		Maintenance	32 (37.65)	66 (57.39)		
		Induction/Consolidation	47 (55.29)	39 (33.91)		
		Relapse/Palliative	6 (7.06)	10 (8.70)		
	Hospitalizations in last 3 months		2.00 (1.00, 2.00)	1.00 (0.00, 2.00)	Z = –3.93	<0.001
	Symptom scores		57.90 (41.60, 71.90)	40.90 (30.00, 58.05)	Z = –3.89	<0.001
Socioeconomic factors						
	Family income (CNY / USD per month)				χ^2^ = 0.38	0.829
		<3000 / <435	23 (27.06)	27 (23.48)		
		3000–8000 / 435–1160	42 (49.41)	61 (53.04)		
		>8000/ >1160	20 (23.53)	27 (23.48)		
	Insurance type				χ^2^ = 0.01	0.993
		Self-pay	7 (8.24)	10 (8.70)		
		Resident insurance	50 (58.82)	67 (58.26)		
		Employee/Commercial	28 (32.94)	38 (33.04)		
	Distance to hospital				χ^2^ = 0.07	0.967
		Local city	25 (29.41)	34 (29.57)		
		Intra-provincial	35 (41.18)	49 (42.61)		
		Inter-provincial	25 (29.41)	32 (27.83)		
	Social support scores		50.00 (45.00, 57.00)	58.00 (52.00, 65.00)	Z = –5.30	<0.001

Continuous variables are presented as median (Q1, Q3). Categorical variables are reported as frequency (percentage). ALL, acute lymphoblastic leukaemia; AML, acute myeloid leukaemia; CNY, Chinese Yuan; USD, United States dollar; Q1, 1st quartile; Q3, 3rd quartile. Currency conversion was based on the March 2026 representative exchange rate of 1 USD = 6.8974 CNY. Converted U.S. dollar values are provided for reference only.

### Factors Associated With Anxiety and Sensitivity Analysis

Multivariable logistic regression analysis identified time since diagnosis, symptom scores, and social support scores as independent factors associated with caregiver anxiety after adjusting for potential confounders. Specifically, for every 6-month increase in time since diagnosis, the risk of caregiver anxiety decreased by 34% (odds ratio (OR) = 0.66, 95% confidence interval (CI): 0.47–0.92). Perceived social support was inversely associated with anxiety, with each 10-point increase in scores significantly linked to a lower likelihood of anxiety (OR = 0.48, 95% CI: 0.32–0.71). Conversely, a heavier symptom burden was associated with greater psychological distress, as each 10-point increase in symptom scores raised the odds of anxiety by 1.22 times (OR = 1.22, 95% CI: 1.03–1.46).

To verify the robustness of these findings, a sensitivity analysis was conducted by replacing symptom scores with the number of hospitalizations in the last three months. The results were consistent with the main model, confirming that time since diagnosis and social support remaining significant predictors. Furthermore, the sensitivity analysis demonstrated that the frequency of hospitalizations in the last three months was an independent risk factor for anxiety (OR = 1.44, 95% CI: 1.08–1.92). Detailed regression results are presented in Table 5 and **Supplementary Table 2**.

**Table 5.** **Multivariable logistic regression for anxiety**.

Variables		Unadjusted		Adjusted		
		β	*p*	β	*p*	OR (95% CI)
Caregiver age (years)		0.03	0.065	0.04	0.066	1.04 (1.00–1.08)
Educational level						
	Junior high school and below					1.00 (Reference)
	High school or equivalent	0.33	0.369	0.35	0.427	1.41 (0.60–3.32)
	College/bachelor’s degree or above	–0.36	0.340	0.02	0.969	1.02 (0.42–2.48)
Child age (years)		0.01	0.872	0.00	0.926	1.00 (0.93–1.09)
Diagnosis						
	ALL					1.00 (Reference)
	AML	–0.07	0.854	–0.36	0.439	0.70 (0.28–1.74)
	Lymphoma	0.14	0.741	–0.32	0.529	0.73 (0.27–1.97)
Time since diagnosis (per 6 months)		–0.43	0.001	–0.42	0.014	0.66 (0.47–0.92)
Treatment stage						
	Maintenance					1.00 (Reference)
	Induction/Consolidation	0.94	0.002	0.05	0.918	1.05 (0.44–2.47)
	Relapse/Palliative	1.20	0.037	0.54	0.451	1.71 (0.42–6.88)
Symptom scores (per 10 points)		0.22	<0.001	0.20	0.023	1.22 (1.03–1.46)
Family income (CNY / USD per month)						
	<3000 / <435					1.00 (Reference)
	3000–8000 / 435–1160	–0.18	0.603	0.04	0.916	1.04 (0.47–2.31)
	>8000/ >1160	0.05	0.896	0.56	0.271	1.76 (0.64–4.80)
Social support scores (per 10 points)		–0.60	<0.001	–0.74	<0.001	0.48 (0.32–0.71)

Adjusted for all variables listed in the table. ALL, acute lymphoblastic leukaemia; AML, acute myeloid leukaemia; CNY, Chinese Yuan; USD, United States dollar; OR, odds ratio; CI, confidence interval. Currency conversion was based on the March 2026 representative exchange rate of 1 USD = 6.8974 CNY. Converted U.S. dollar values are provided for reference only.

### Factors Associated With Depression and Sensitivity Analysis

Multivariable logistic regression analysis identified educational level, marital status, symptom scores, and social support scores as independent predictors of caregiver depression. Caregivers with a college degree or above had a significantly lower risk of depression compared to those with a junior high school education or below (OR = 0.34, 95% CI: 0.13–0.89). Marital status was also a key predictor, with divorced, widowed, or unmarried caregivers having 3.97 times the odds of depression compared to married caregivers (OR = 3.97, 95% CI: 1.35–11.69). Regarding clinical and psychosocial factors, every 10-point increase in symptom scores was associated with a 1.55-fold increase in the likelihood of depression (OR = 1.55, 95% CI: 1.25–1.91), while every 10-point increase in social support scores reduced the risk of depression by 69% (OR = 0.31, 95% CI: 0.19–0.50). Notably, time since diagnosis and treatment stage, which were significant in the univariate analysis, did not retain statistical significance after adjusting for confounders.

The sensitivity analysis further supported the robustness of these findings. When symptom scores were replaced with the number of hospitalizations in the preceding three months, social support remained a significant independent predictor. Additionally, a higher frequency of recent hospitalizations was associated with an increased risk of depression (OR = 1.47, 95% CI: 1.11–1.95). Detailed results are shown in Table 6 and **Supplementary Table 3**.

**Table 6.** **Multivariable logistic regression for depression**.

Variables		Unadjusted		Adjusted		
		β	*p*	β	*p*	OR (95% CI)
Sex						
	Male					1.00 (Reference)
	Female	–0.50	0.102	–0.64	0.118	0.53 (0.24–1.18)
Educational level						
	Junior high school and below					1.00 (Reference)
	High school or equivalent	0.15	0.675	–0.00	0.994	1.00 (0.40–2.46)
	College/bachelor’s degree or above	–1.06	0.007	–1.07	0.029	0.34 (0.13–0.89)
Marital status						
	Married					1.00 (Reference)
	Divorced/Widowed/Unmarried	0.89	0.039	1.38	0.012	3.97 (1.35–11.69)
Child age (years)		0.04	0.292	0.07	0.139	1.07 (0.98–1.17)
Diagnosis						
	ALL					1.00 (Reference)
	AML	0.55	0.164	–0.23	0.662	0.79 (0.28–2.23)
	Lymphoma	0.22	0.600	–0.94	0.102	0.39 (0.13–1.20)
Time since diagnosis (months)		–0.38	0.005	–0.31	0.092	0.73 (0.51–1.05)
Treatment stage						
	Maintenance					1.00 (Reference)
	Induction/Consolidation	0.91	0.003	–0.03	0.944	0.97 (0.37–2.53)
	Relapse/Palliative	0.21	0.703	–1.29	0.100	0.28 (0.06–1.28)
Symptom scores		0.25	<0.001	0.44	<0.001	1.55 (1.25–1.91)
Family income (CNY / USD per month)						
	<3000 / <435					1.00 (Reference)
	3000–8000 / 435–1160	–0.21	0.540	0.04	0.936	1.04 (0.43–2.51)
	>8000/ >1160	–0.14	0.733	0.79	0.175	2.20 (0.71–6.84)
Social support scores		–0.95	<0.001	–1.18	<0.001	0.31 (0.19–0.50)

Adjusted for all variables listed in the table. ALL, acute lymphoblastic leukaemia; AML, acute myeloid leukaemia; CNY, Chinese Yuan; USD, United States dollar; OR, odds ratio; CI, confidence interval. Currency conversion was based on the March 2026 representative exchange rate of 1 USD = 6.8974 CNY. Converted U.S. dollar values are provided for reference only.

### Diagnostic Testing and Performance of the Anxiety and Depression

Comprehensive diagnostic tests were performed for the multivariable logistic regression model of anxiety. Collinearity analysis showed that the VIF for all included variables ranged from 1.030 to 1.680, with tolerance values exceeding 0.59, indicating the absence of significant multicollinearity among the independent variables. The Hosmer-Lemeshow test yielded a non-significant result (χ^2^ = 10.177, *p* = 0.253), suggesting that the model demonstrated a good fit to the observed data. Furthermore, ROC curve analysis indicated that the model possessed good discrimination capability, with an area AUC of 0.773. Detailed diagnostic metrics and the ROC curve are presented in **Supplementary Material 1**.

**Supplementary Material 2** presents the collinearity diagnostics, goodness-of-fit, and discrimination performance of the multivariable logistic regression model for caregiver depression. The variance inflation factors ranged from 1.030 to 1.690 and tolerance values ranged from 0.592 to 0.971, indicating no notable multicollinearity. The Hosmer-Lemeshow test supported good model fit with χ^2^ = 4.644, df = 8, and *p* = 0.795. The ROC curve suggested good discrimination, with an AUC of 0.839.

## Discussion

This study investigated the prevalence of anxiety and depression among primary caregivers of children with haematological malignancies and analysed associated factors. The results indicated that more than half of the caregivers experienced anxiety symptoms, and approximately forty percent experienced depression. This prevalence is notably higher than that observed in the general population and aligns with global data regarding psychological distress among parents of children with cancer [8]. These findings suggest that primary caregivers bear a substantial psychological burden throughout the prolonged disease course, representing a pervasive clinical challenge that warrants significant attention from healthcare professionals.

Regarding factors associated with anxiety, the multivariable model highlighted the distinct magnitude of association for each independent factor. Time since diagnosis was inverse association (OR = 0.66), suggesting that for every 6-month progression, the odds of anxiety significantly decrease as caregivers gradually adapt to the clinical environment. Conversely, symptom burden showed a positive association (OR = 1.22), indicating that even modest increases in the child’s physical suffering substantially elevate the likelihood of caregiver anxiety. Social support emerged as the strongest inversely associated factor (OR = 0.48), where higher perceived support was associated with a more than 50 percent reduction in the risk of anxiety. These regression coefficients underscore that acute clinical stressors and psychosocial resources are the primary correlates of the anxiety profile in this population. This finding is likely attributable to diagnostic uncertainty and unfamiliarity with caregiving tasks during the initial phase. As treatment progresses, caregivers appear to adapt to disease-related stress, leading to a stabilization of their psychological status [22]. Symptom burden was identified as a common risk factor for both anxiety and depression; this finding supports previous evidence suggesting that a child’s physical suffering is closely linked to caregiver’s psychological distress [23]. When children experience frequent or severe symptoms, caregivers often feel helpless due to their inability to effectively alleviate the child’s pain, thereby intensifying their own distress. Furthermore, perceived social support demonstrated a significant protective effect, reaffirming the “stress-buffering hypothesis” in social psychology [24]. Support from family, friends, and the medical team provides emotional reassurance and practical resources, effectively buffering the strain of long-term caregiving.

For depressive symptoms, educational level and marital status were identified as independent predictors in the main model [25]. Notably, variables such as treatment stage and time since diagnosis were significant in the univariable analysis but did not retain significance in the multivariable model for depression. This phenomenon suggests that the influence of these clinical factors may be partially overlapping or mediated by more proximal stressors including child symptom burden and perceived social support. Consistent with previous evidence linking educational attainment to health literacy and mental health, our results suggest that caregivers with higher educational attainment may be better able to obtain and understand health information, which may be associated with better psychological outcomes [26, 27]. Conversely, divorced or widowed caregivers face a significantly increased risk of depression, likely due to the lack of spousal emotional support [28]. This finding implies that clinical practice should prioritize vulnerable groups with weak social support systems and lower educational levels.

To evaluate the robustness of the results, a sensitivity analysis was conducted. Given the intrinsic correlation between symptom burden and hospitalization frequency—where severe or frequent symptoms typically necessitate more hospital admissions—we substituted symptom scores with the frequency of recent hospitalizations in the model [16]. In the sensitivity analysis, more frequent hospitalizations in the preceding three months remained associated with higher odds of caregiver anxiety and depression. This finding is clinically plausible, as parents of children with cancer and parents of hospitalized children commonly experience substantial psychological distress during intensive care periods [9, 29]. Notably, in the sensitivity analysis, while the protective effect of social support remained robust, educational level exhibited only a borderline trend (*p* = 0.058), and marital status was no longer statistically significant. This suggests that, compared with relatively may have a more somewhat variable demographic features, clinical situational factors and psychosocial resources show a more central and stable association with caregiver depression [30].

Our findings underscore the need for structured social support interventions within clinical settings [31]. Peer support programs may be considered, as parents often value emotional support and practical guidance from peers with similar caregiving experiences [32]. Structured psychoeducational sessions may also be integrated into nursing care to address caregivers’ informational and psychosocial needs and to help reduce anxiety [33]. For caregivers with greater psychosocial vulnerability, timely referral to professional psychosocial services and additional family-centred support may be warranted [34]. Utilizing digital platforms to host support forums can further extend these resources to families living far from the hospital.

Several limitations of this study should be acknowledged. First, the cross-sectional nature of this study precludes the establishment of causal inference. The observed associations between variables should be interpreted as correlates rather than evidence of causality. Future longitudinal studies should be conducted to track the evolution of psychological distress and clarify causal pathways. Second, this study used convenience sampling at a single tertiary hospital, which may lead to selection bias. Caregivers in such specialized settings often manage children with higher symptom burdens or more advanced disease stages compared to those in outpatient departments. This limits the generalizability of our results to caregivers in non-tertiary medical centres or community-based settings. Future multi-centre studies involving diverse clinical environments are necessary to better understand the mental health needs of the broader caregiver population. Third, regarding the assessment of child symptom burden, we employed a uniform caregiver-proxy report method for all age groups instead of using age-specific self-report versions. While this ensured consistency in measuring the caregivers’ perception of stress, it may differ from the children’s actual symptom experience. Future studies could incorporate both perspectives for a more comprehensive assessment. Fourth, although multiple confounders were controlled for, unmeasured variables such as personality traits, history of mental illness, or genetic background were not included. Future studies should incorporate these variables to refine the risk models. Finally, the single time-point assessment does not capture the dynamic trajectory of caregiver mental health throughout the treatment process. Implementing prospective designs with multiple measurement points throughout the treatment course will be essential to identify critical windows for psychological interventions.

## Conclusions

Primary caregivers of children with haematological malignancies face a prevalent risk of experiencing high levels of anxiety and depression. The early post-diagnosis period, heavy symptom burden of the child, and the lack of social support are independent factors associated with psychological distress. Although educational level and marital status showed associations with depression, sensitivity analysis suggests their influence may be contextual to the clinical setting. These findings underscore the critical importance of integrating caregiver mental health into routine clinical assessment. Future intervention strategies should focus on strengthening psychological support during the early diagnostic phase, optimizing symptom management for children, and facilitating the establishment of multi-dimensional social support systems to improve the overall quality of life for both caregivers and patients.

## Availability of Data and Materials

All experimental data included in this study can be obtained by contacting the corresponding author if needed.
