# Supplementary material for: Prevalence and Associated Factors of Anxiety and Depression Among Primary Caregivers of Children With Haematological Malignancies: A Cross-sectional Study
Source: Actas Esp Psiquiatr. 2026 Apr 15;54(2):419–31. doi: 10.62641/aep.v54i2.2195 (PMC13180661; doi:10.62641/aep.v54i2.2195)
Supplement: Supplementary file 1 [file ActEsp-54-2-419-431-s1.zip › Supplementary Material 1.docx]

| Supplementary Table 4 Collinearity diagnostics for variables in the multivariable logistic regression model of anxiety | | |
| --- | --- | --- |
| Variables | VIF | Tolerance |
| Caregiver age (years) | 1.131 | 0.884 |
| Educational level | 1.220 | 0.820 |
| Child age (years) | 1.030 | 0.971 |
| Diagnosis | 1.208 | 0.827 |
| Time since diagnosis (per 6 months) | 1.193 | 0.838 |
| Treatment stage | 1.680 | 0.595 |
| Symptom scores (per 10 points) | 1.600 | 0.625 |
| Family income (CNY / month) | 1.226 | 0.816 |
| Social support scores (per 10 points) | 1.182 | 0.846 |

VIF, variance inflation factor; CNY, Chinese Yuan.

| Supplementary Table 5 Hosmer-Lemeshow goodness-of-fit test for the anxiety model | | |
| --- | --- | --- |
| χ^2^ | df | *P* |
| 10.177 | 8 | 0.253 |

df, degrees of freedom.

Supplementary Figure 1 ROC curve for the multivariable logistic regression model predicting caregiver anxiety


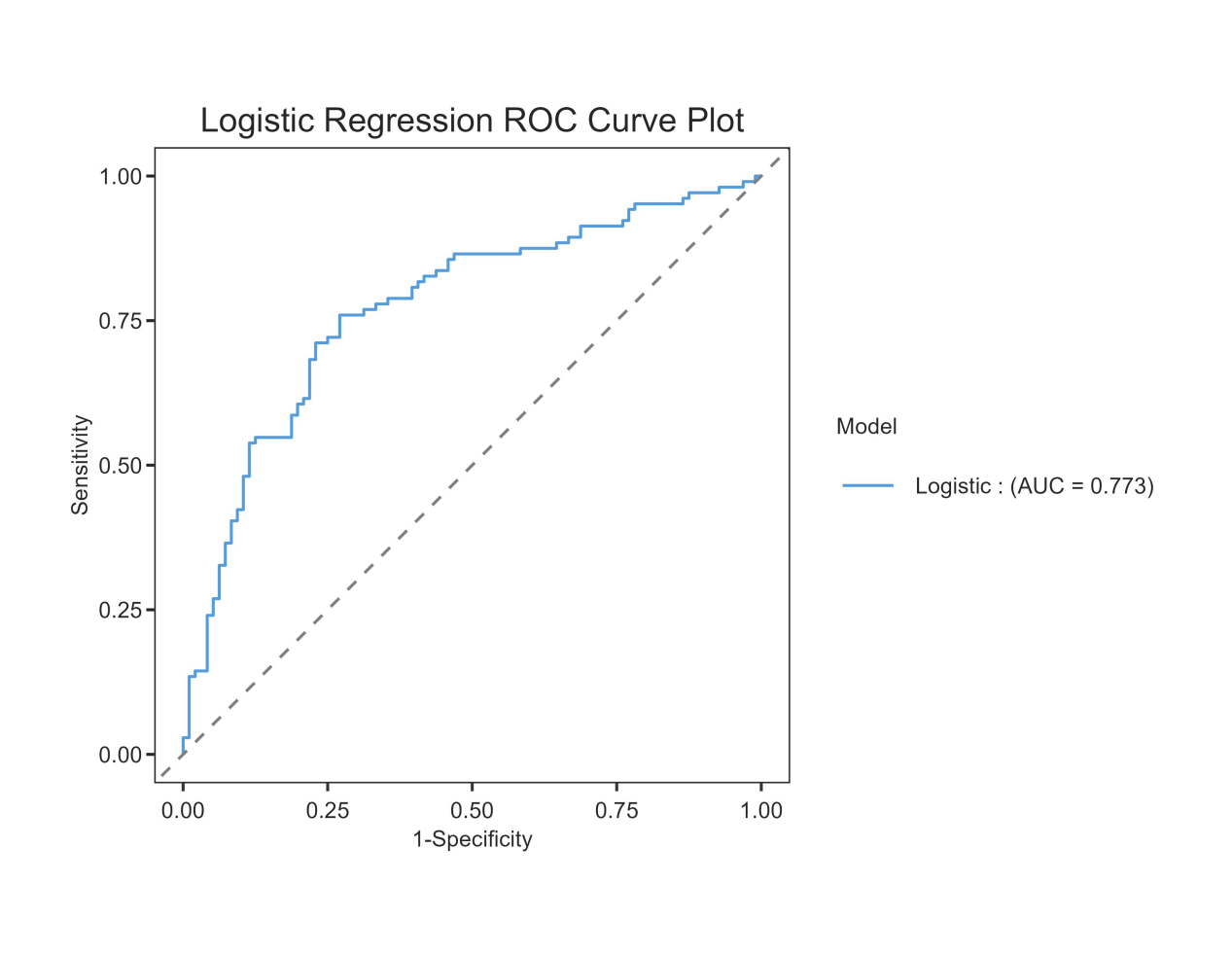


ROC, receiver operating characteristic; AUC, area under the curve.
