# Supplementary material for: Prevalence and Associated Factors of Anxiety and Depression Among Primary Caregivers of Children With Haematological Malignancies: A Cross-sectional Study
Source: Actas Esp Psiquiatr. 2026 Apr 15;54(2):419–31. doi: 10.62641/aep.v54i2.2195 (PMC13180661; doi:10.62641/aep.v54i2.2195)
Supplement: Supplementary file 1 [file ActEsp-54-2-419-431-s1.zip › Supplementary Material 2.docx]

| Supplementary Table 6 Collinearity diagnostics for variables in the multivariable logistic regression model of depression | | |
| --- | --- | --- |
| Variables | VIF | Tolerance |
| Sex | 1.071 | 0.934 |
| Educational level | 1.167 | 0.857 |
| Marital status | 1.065 | 0.939 |
| Child age (years) | 1.030 | 0.971 |
| Diagnosis | 1.231 | 0.813 |
| Time since diagnosis (per 6 months) | 1.192 | 0.839 |
| Treatment stage | 1.690 | 0.592 |
| Symptom scores (per 10 points) | 1.671 | 0.599 |
| Family income (CNY / month) | 1.206 | 0.829 |
| Social support scores (per 10 points) | 1.205 | 0.830 |

VIF, variance inflation factor; CNY, Chinese Yuan.

| Supplementary Table 7 Hosmer-Lemeshow goodness-of-fit test for the depression model | | |
| --- | --- | --- |
| χ^2^ | df | *P* |
| 4.644 | 8 | 0.795 |

df, degrees of freedom.

Supplementary Figure 2 ROC curve for the multivariable logistic regression model predicting caregiver depression


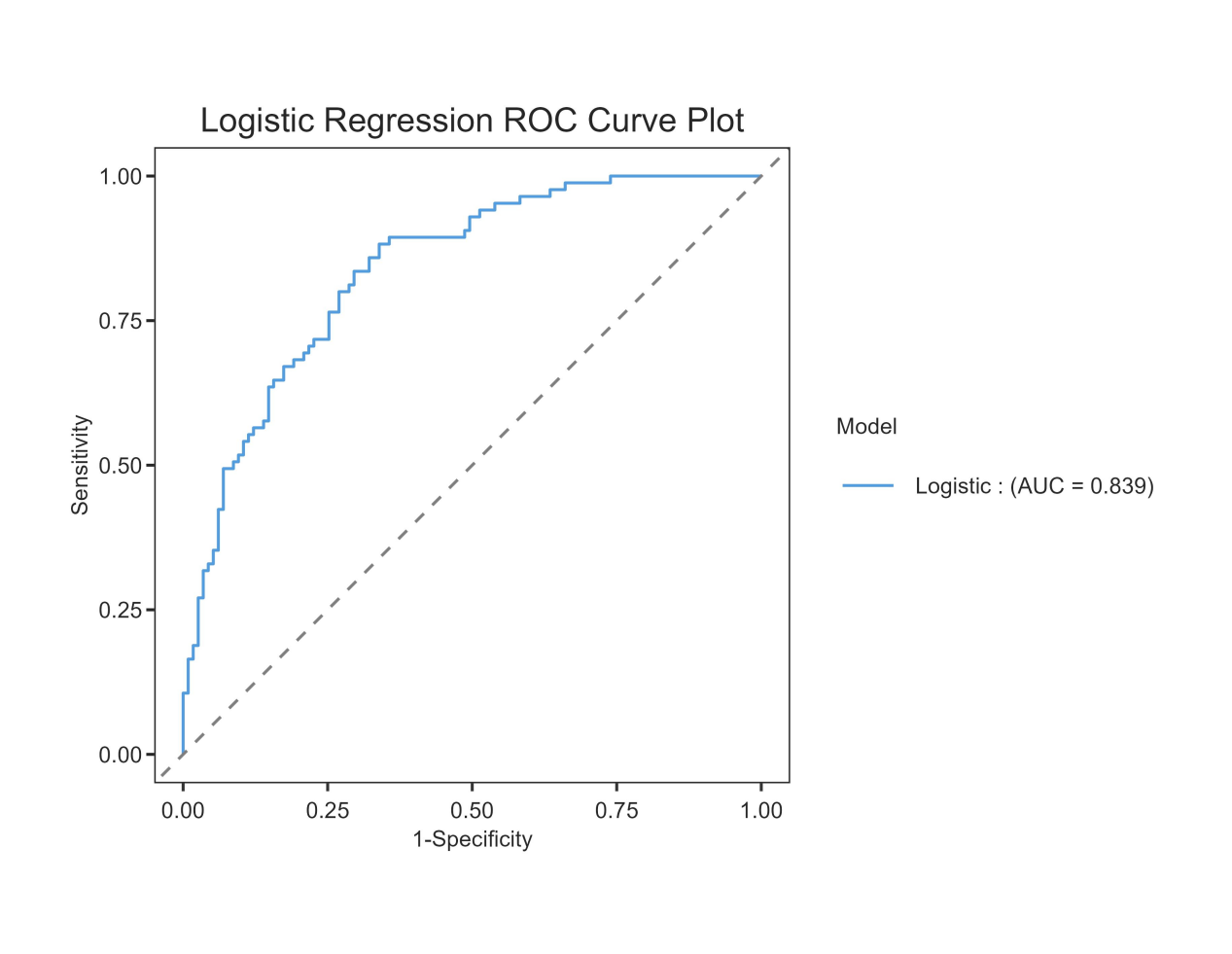


ROC, receiver operating characteristic; AUC, area under the curve.
