# Supplementary material for: Prevalence and Associated Factors of Anxiety and Depression Among Primary Caregivers of Children With Haematological Malignancies: A Cross-sectional Study
Source: Actas Esp Psiquiatr. 2026 Apr 15;54(2):419–31. doi: 10.62641/aep.v54i2.2195 (PMC13180661; doi:10.62641/aep.v54i2.2195)
Supplement: Supplementary file 1 [file ActEsp-54-2-419-431-s1.zip › Supplementary Table 1.docx]

Supplementary Table 1 Reliability and validity analysis of the HADS

|  | **No. of Items** | **N** | **Cronbach’s Alpha** |
| --- | --- | --- | --- |
| **HADS-A** | 7 | 200 | 0.944 |
| **HADS-D** | 7 | 200 | 0.917 |

HADS, Hospital Anxiety and Depression Scale; HADS-A, Anxiety of the Hospital Anxiety and Depression Scale; HADS-D, Depression of the Hospital Anxiety and Depression Scale.
