# Supplementary material for: Prevalence and Associated Factors of Anxiety and Depression Among Primary Caregivers of Children With Haematological Malignancies: A Cross-sectional Study
Source: Actas Esp Psiquiatr. 2026 Apr 15;54(2):419–31. doi: 10.62641/aep.v54i2.2195 (PMC13180661; doi:10.62641/aep.v54i2.2195)
Supplement: Supplementary file 1 [file ActEsp-54-2-419-431-s1.zip › Supplementary Table 2.docx]

Supplementary Table 2 Multivariable logistic regression for anxiety (sensitivity analysis)

| Variables | Unadjusted | |  | Adjusted | | |
| --- | --- | --- | --- | --- | --- | --- |
|  | β | *P* |  | β | *P* | OR (95%CI) |
| Caregiver age (years) | 0.03 | 0.065 |  | 0.03 | 0.106 | 1.03 (0.99 ~ 1.07) |
| Educational level |  |  |  |  |  |  |
| Junior high school and below |  |  |  |  |  | 1.00 (Reference) |
| High school or equivalent | 0.33 | 0.369 |  | 0.39 | 0.371 | 1.48 (0.63 ~ 3.49) |
| College / Bachelor degree or above | -0.36 | 0.340 |  | 0.05 | 0.905 | 1.06 (0.43 ~ 2.57) |
| Child age (years) | 0.01 | 0.872 |  | -0.00 | 0.969 | 1.00 (0.92 ~ 1.08) |
| Diagnosis |  |  |  |  |  |  |
| ALL |  |  |  |  |  | 1.00 (Reference) |
| AML | -0.07 | 0.854 |  | -0.14 | 0.752 | 0.87 (0.35 ~ 2.12) |
| Lymphoma | 0.14 | 0.741 |  | -0.19 | 0.709 | 0.83 (0.31 ~ 2.20) |
| Time since diagnosis (months) | -0.43 | 0.001 |  | -0.41 | **0.018** | 0.67 (0.48 ~ 0.93) |
| Treatment stage |  |  |  |  |  |  |
| Maintenance |  |  |  |  |  | 1.00 (Reference) |
| Induction / Consolidation | 0.94 | 0.002 |  | 0.17 | 0.675 | 1.19 (0.53 ~ 2.65) |
| Relapse / Palliative | 1.20 | 0.037 |  | 0.84 | 0.210 | 2.31 (0.63 ~ 8.50) |
| Hospitalizations in last 3 months | 0.43 | < 0.001 |  | 0.36 | **0.014** | 1.44 (1.08 ~ 1.92) |
| Family income (CNY / month) |  |  |  |  |  |  |
| < 3000 |  |  |  |  |  | 1.00 (Reference) |
| 3000-8000 | -0.18 | 0.603 |  | 0.01 | 0.983 | 1.01 (0.45 ~ 2.24) |
| > 8000 | 0.05 | 0.896 |  | 0.62 | 0.225 | 1.86 (0.68 ~ 5.07) |
| Social support scores | -0.60 | < 0.001 |  | -0.72 | **< 0.001** | 0.49 (0.33 ~ 0.73) |

Symptom scores were replaced by the number of hospitalizations in the past 3 months to test model robustness. Adjusted for all variables listed in the table. ALL, acute lymphoblastic leukemia; AML, acute myeloid leukemia; CNY, Chinese Yuan; OR, odds ratio; CI, confidence interval.
