# Supplementary material for: Prevalence and Associated Factors of Anxiety and Depression Among Primary Caregivers of Children With Haematological Malignancies: A Cross-sectional Study
Source: Actas Esp Psiquiatr. 2026 Apr 15;54(2):419–31. doi: 10.62641/aep.v54i2.2195 (PMC13180661; doi:10.62641/aep.v54i2.2195)
Supplement: Supplementary file 1 [file ActEsp-54-2-419-431-s1.zip › Supplementary Table 3.docx]

Supplementary Table 3 Multivariable logistic regression for depression (sensitivity analysis)

| Variables | Unadjusted | |  | Adjusted | | |
| --- | --- | --- | --- | --- | --- | --- |
|  | β | *P* |  | β | *P* | OR (95%CI) |
| Sex |  |  |  |  |  |  |
| Male |  |  |  |  |  | 1.00 (Reference) |
| Female | -0.50 | 0.102 |  | -0.37 | 0.344 | 0.69 (0.32 ~ 1.48) |
| Educational level |  |  |  |  |  |  |
| Junior high school and below |  |  |  |  |  | 1.00 (Reference) |
| High school or equivalent | 0.15 | 0.675 |  | 0.07 | 0.874 | 1.07 (0.44 ~ 2.60) |
| College / Bachelor degree or above | -1.06 | 0.007 |  | -0.90 | 0.058 | 0.41 (0.16 ~ 1.03) |
| Marital status |  |  |  |  |  |  |
| Married |  |  |  |  |  | 1.00 (Reference) |
| Divorced / Widowed / Unmarried | 0.89 | 0.039 |  | 0.81 | 0.120 | 2.24 (0.81 ~ 6.17) |
| Child age (years) | 0.04 | 0.292 |  | 0.05 | 0.243 | 1.05 (0.97 ~ 1.15) |
| Diagnosis |  |  |  |  |  |  |
| ALL |  |  |  |  |  | 1.00 (Reference) |
| AML | 0.55 | 0.164 |  | 0.37 | 0.471 | 1.44 (0.53 ~ 3.89) |
| Lymphoma | 0.22 | 0.600 |  | -0.46 | 0.391 | 0.63 (0.22 ~ 1.81) |
| Time since diagnosis (months) | -0.38 | 0.005 |  | -0.23 | 0.196 | 0.79 (0.56 ~ 1.13) |
| Treatment stage |  |  |  |  |  |  |
| Maintenance |  |  |  |  |  | 1.00 (Reference) |
| Induction / Consolidation | 0.91 | 0.003 |  | 0.52 | 0.238 | 1.69 (0.71 ~ 4.03) |
| Relapse / Palliative | 0.21 | 0.703 |  | -0.38 | 0.607 | 0.69 (0.16 ~ 2.89) |
| Hospitalizations in last 3 months | 0.37 | 0.001 |  | 0.39 | **0.007** | 1.47 (1.11 ~ 1.95) |
| Family income (CNY / month) |  |  |  |  |  |  |
| < 3000 |  |  |  |  |  | 1.00 (Reference) |
| 3000-8000 | -0.21 | 0.540 |  | 0.01 | 0.985 | 1.01 (0.42 ~ 2.42) |
| > 8000 | -0.14 | 0.733 |  | 0.78 | 0.162 | 2.18 (0.73 ~ 6.49) |
| Social support scores | -0.95 | < 0.001 |  | -1.08 | **< 0.001** | 0.34 (0.21 ~ 0.54) |

Symptom scores were replaced by the number of hospitalizations in the past 3 months to test model robustness. Adjusted for all variables listed in the table. ALL, acute lymphoblastic leukemia; AML, acute myeloid leukemia; CNY, Chinese Yuan; OR, odds ratio; CI, confidence interval.
